# Combined effect of hypertension and hyperuricemia on ischemic stroke in a rural Chinese population

**DOI:** 10.1186/s12889-021-10858-x

**Published:** 2021-04-23

**Authors:** Peng Sun, Mengqi Chen, Xiaofan Guo, Zhao Li, Ying Zhou, Shasha Yu, Hongmei Yang, Guozhe Sun, Liqiang Zheng, Yingxian Sun

**Affiliations:** 1 Department of Ophthalmology, the First Hospital of China Medical University, Shenyang, Liaoning China; 2 Department of Cardiology, the First Hospital of China Medical University, 155 Nanjing North Street, Heping District, Shenyang, 110001 Liaoning China; 3 Department of Clinical Epidemiology, Library, Shengjing Hospital of China Medical University, Shenyang, Liaoning China

**Keywords:** Hypertension, Hyperuricemia, Ischemic stroke

## Abstract

**Background:**

To investigate the combined effect of hypertension and hyperuricemia to the risk of ischemic stroke in a rural Chinese population.

**Methods:**

The cross-sectional study was conducted from 2012 to 2013 in a rural area of China. After exclusion for missing data, we finally included 11,731 participants into analysis.

**Results:**

After adjusting for age, current smoking, current drinking, BMI, TG, HDL-C and eGFR, hypertension was significantly associated with ischemic stroke in men (OR: 2.783, 95% CI: 1.793, 4.320) and in women (OR: 4.800, 95% CI: 2.945, 7.822). However, hyperuricemia was significantly associated with ischemic stroke only in women (OR: 1.888, 95% CI: 1.244, 2.864). After full adjustment, participants with both hypertension and hyperuricemia had 8.9 times higher risk than those without them. Finally, the interaction between hypertension and hyperuricemia was statistically significant only in women rather than in men after full adjustment.

**Conclusions:**

This study demonstrated the positive correlations between hypertension, hyperuricemia and ischemic stroke. Our study also demonstrated the joint effect between hypertension and hyperuricemia towards ischemic stroke only in women, not in men.

## Background

Globally, stroke is a major cause of death and adult disability [1]. By 2013, 27 of China’s 33 provinces had stroke as the main cause of death [2]. In China, the annual stroke mortality rate is about 1.6 million people, which is about 157 deaths per 100,000 people due to stroke [3]. Among 100,000 people, strokes caused about 116 deaths in urban areas and 111 deaths in rural areas [3]. Therefore, stroke has emerged as a major health problem in China.

Hypertension (HTN) is one of the important risk factors of stroke. More than 60% of acute stroke patients had elevated blood pressure [4]. Furthermore, more than 70% of stroke patients had a history of hypertension, and nearly half of them had poor baseline blood pressure control [5–7]. The incidence of stroke increased proportionally with the increase in systolic and diastolic blood pressure, with a 3.1-fold increase in the relative risk for men and a 2.9-fold increase in women [8, 9]. Hyperuricemia is another potential important risk factor of stroke. The elevated level of serum uric acid was independently positively correlated with ischemic stroke in patients with aged < 60 years [10]. Previous prospective observational studies have showed that hyperuricemia was independently correlated with stroke incidence and mortality [11, 12]. On the other hand, hyperuricemia was also associated with hypertension, type 2 diabetes, dyslipidemia, chronic kidney disease, and cardiovascular events, particularly stroke [13–15].

Previous studies have clarified the potential correlation between hypertension, hyperuricemia and ischemic stroke. However, these studies only examined the independent effects of risk factors. So far, no studies have investigated the combined effect of hypertension and hyperuricemia to ischemic stroke. Thus, this study aimed to investigate the combined effect of HTN and hyperuricemia to the risk of ischemic stroke.

## Methods

### Study population

The present study was based on a cross-sectional epidemiological survey known as NCRCHS which conducted from January 2012 to August 2013. The detailed design and rationale of NCRCHS were fully discussed elsewhere [16]. A total number of 11,956 participants (age ≥ 35 years) were collected from Liaoning province, northeastern China. In the present work, 225 participants were further excluded for missing data. Eventually, 11,731 subjects were enrolled into the present work. Our study was approved by the Ethics Committee of China Medical University (Shenyang, China). All subjects provided written informed consent.

### Data collection and measurements

The process about data collection and measurements was fully described in our previously published studies [17]. Before the survey, cardiologists and nurses participated a professional training, passed a standardized exam, and acquired the qualification to gather data. The data were collected through uniform questionnaires regarding demographic data, anthropometric parameters, health-related behaviors. The central steering committee with a subcommittee conducted the quality assurance process of data collection. The questionnaire was designed to collect detail information from participants. Smoking and drinking status were separated into current status and others according to participants’ self-reports. After participants rested for at least five minutes in a properly relaxed and sitting state, the blood pressure was taken three times and measured by two randomly selected workers. The mean reading of three consecutive values was determined as the final result of blood pressure. Before the measurement of anthropometric indices, lightweight clothes without shoes were required for the subjects. The weight of participants was quantified to 0.1 kg by calibrated digital scales and height was quantified to 0.1 cm by portable stadiometers. After individuals were fasting at least 12 h, blood samples were collected from the antecubital veins in the morning. For long-term storage, the serum of venous blood sample was isolated by calibrated centrifuge and frozen at − 20 °C. The fasting blood samples of individuals were tested by Olympus AU640 auto analyzers to measure the blood concentrations of FPG, TG, HDL-C, Scr and SUA.

### Definition

The body mass index (BMI) was determined as: weight (kg)/height (m^2^). The estimated glomerular filtration rate (eGFR) was defined according to the CKD-EPI equation [18]. The definition of hyperuricemia was serum uric acid (SUA) ≥357 μmol/L for females and ≥ 417 μmol/L for males [19]. Hypertension was determined as systolic blood pressure (SBP) ≥140 mmHg and/or diastolic blood pressure (DBP) ≥90 mmHg [20]. Ischemic stroke was determined as a history of cerebrovascular events, which was demonstrated by either cranial CT or MR scan within the past 2 years.

### Statistical analysis

The results were displayed as mean values ± standard deviation (SD) or median (interquartile) for continuous variables. The following category variables were presented as frequencies (percentages). Students’ t-test or Mann-Whitney test was applied to compare continuous variables between groups. The chi-square test was performed to compare category variables between groups. Additionally, the rank-sum test was used to compare ordinal category variables between groups. Multivariate logistic regression analyses were performed to evaluate the relationship of hypertension and hyperuricemia to ischemic stroke. The results were displayed as odds ratio (OR) and 95% confidence interval (95% CI). All of the analyses were performed by SPSS 25.0 software (IBP corp). A two-tailed *P* value < 0.05 was considered as significant.

## Results

**Fig. 1:**
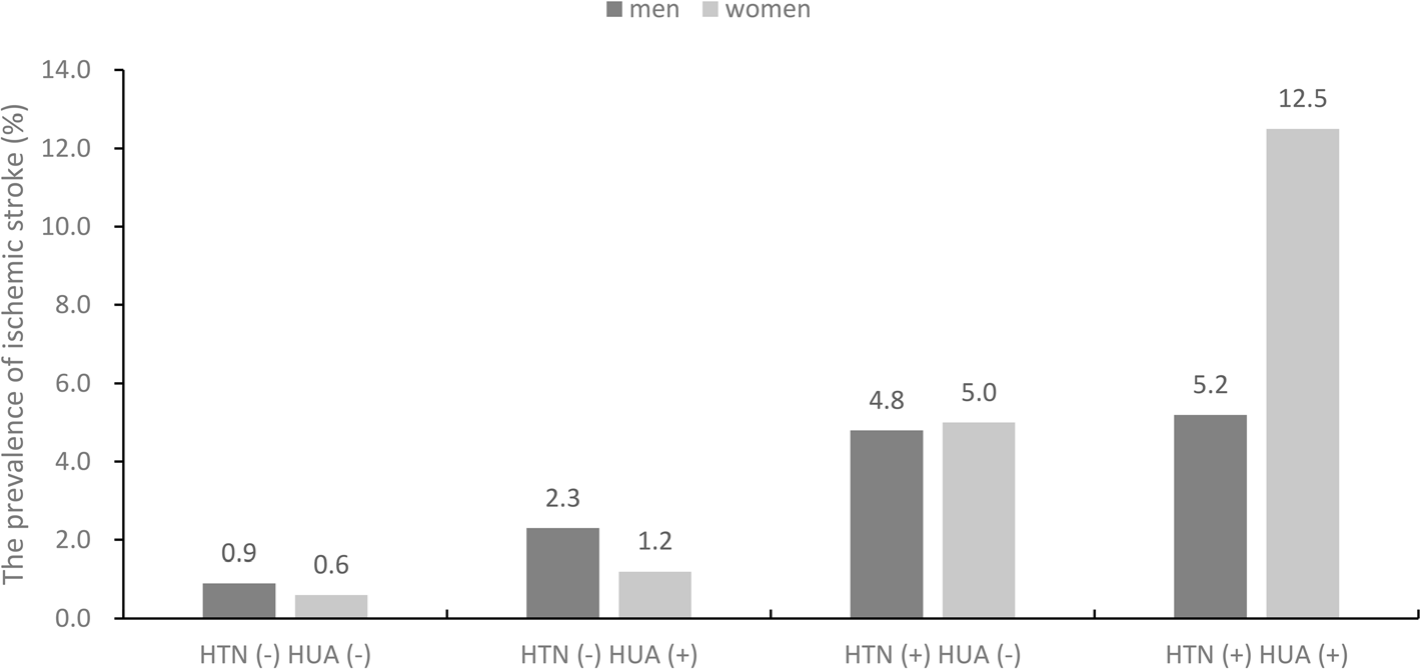
Prevalence of ischemic stroke according to the hypertension and hyperuricemia stratified by sex

Table 1 shows the characteristics of study population divided by ischemic stroke and sex. The prevalence of ischemic stroke was 3.16% in men and 3.12% in women. Population with ischemic stroke had higher age, SBP and DBP in both sexes, and had higher BMI, TG and SUA only in women. The percentage of current smoking and current drinking was dramatically lower in patients group than those in normal group in both sexes. The percentage of hyperuricemia was higher only in women and hypertension was higher in both genders. As shown in Fig. 1, the prevalence of ischemic stroke was greater in HTN (+) and HUA (+) in both gender than in HTN (−) and HUA (−) (0.9% vs. 5.2% for male; 0.6% vs. 12.5% for female).

**Table 1:** Characteristics of study population divided by ischemic stroke and sex

	Men			Women		
	Ischemic stroke			Ischemic stroke		
	No (*n* = 5206)	Yes (*n* = 170)	*p* value	No (*n* = 6104)	Yes (*n* = 197)	*p* value
Age (years)	54.0 ± 10.7	63.7 ± 9.1	< 0.001	53.1 ± 10.3	62.9 ± 8.2	< 0.001
Current smoking (%)	3000 (57.0)	81 (47.6)	0.015	1002 (16.4)	30 (15.2)	0.658
Current drinking (%)	2405 (45.7)	44 (25.9)	< 0.001	183 (3.0)	0 (0.0)	0.014
BMI (kg/m^2^)	24.7 ± 3.5	25.0 ± 3.4	0.362	24.8 ± 3.8	26.0 ± 3.7	< 0.001
FPG (mmol/L)	5.6 (5.2–6.1)	5.8 (5.4–6.4)	< 0.001	5.5 (5.1–6.0)	5.7 (5.3–6.7)	< 0.001
TC (mmol/L)	5.2 ± 1.0	5.2 ± 1.0	0.586	5.3 ± 1.1	5.6 ± 1.0	< 0.001
TG (mmol/L)	1.2 (0.9–1.9)	1.4 (1.0–2.1)	0.055	1.2 (0.9–1.9)	1.9 (1.2–2.8)	< 0.001
HDL-C (mmol/L)	1.4 ± 0.4	1.3 ± 0.4	0.012	1.4 ± 0.3	1.3 ± 0.3	< 0.001
LDL-C (mmol/L)	2.9 ± 0.8	3.0 ± 0.8	0.004	3.0 ± 0.8	3.3 ± 0.8	< 0.001
eGFR (ml/min/1.73 m^2^)	94.5 ± 15.3	85.5 ± 15.3	< 0.001	92.4 ± 16.1	82.6 ± 18.7	< 0.001
SBP (mmHg)	143.2 ± 22.2	159.8 ± 26.9	< 0.001	139.5 ± 23.6	161.3 ± 27.4	< 0.001
DBP (mmHg)	83.6 ± 11.8	87.7 ± 11.8	< 0.001	80.4 ± 11.5	85.8 ± 12.2	< 0.001
SUA (μmol/L)	333.3 ± 83.0	344.4 ± 97.5	0.088	254.6 ± 66.7	289.8 ± 88.6	< 0.001
Hyperuricemia (%)	779 (14.8)	33 (19.4)	0.098	432 (7.1)	41 (20.8)	< 0.001
Hypertension (%)	2787 (53.0)	143 (84.1)	< 0.001	2889 (47.3)	177 (89.8)	< 0.001
Anti-hypertensive drug	612 (11.6)	89 (52.4)	< 0.001	956 (15.6)	127 (64.5)	< 0.001

Data are expressed as mean ± standard deviation (SD) or median (interquartile range) and numbers (percentage) as appropriate

*Abbreviations*: *BMI* body mass index, *FPG* fasting plasma glucose, *TC* total cholesterol, *TG* triglyceride, *HDL-C* high-density lipoprotein cholesterol, *LDL-C* low-density lipoprotein cholesterol, *eGFR* estimated glomerular filtration rate, *SBP* systolic blood pressure, *DBP* diastolic blood pressure, *SUA* serum uric acid

Logistic regression was performed to display the association of SBP, DBP and SUA with ischemic stroke by sex (Table 2). After adjusting for age, current smoking, current drinking, BMI, FPG, TC, TG, HDL-C, LDL-C and eGFR, SBP was significantly associated with ischemic stroke in men (OR: 1.017, 95% CI: 1.011, 1.024) and in women (OR: 1.018, 95% CI: 1.013, 1.024). Similarly, DBP and SUA was significantly associated with ischemic stroke in both men and women.

**Table 2:** Multivariate logistic regression of SBP, DBP and SUA for ischemic stroke by sex

	Odds Ratio (95%CI)					
	Model 1	*P* value	Model 2	*P* value	Model 3	*P* value
Men						
SBP	1.027 (1.021, 1.033)	< 0.001	1.020 (1.013, 1.026)	< 0.001	1.017 (1.011, 1.024)	< 0.001
DBP	1.027 (1.015, 1.039)	< 0.001	1.035 (1.022, 1.048)	< 0.001	1.032 (1.018, 1.045)	< 0.001
SUA	1.002 (1.000, 1.003)	0.087	1.002 (1.001, 1.004)	0.008	1.002 (1.000, 1.004)	0.032
Women						
SBP	1.030 (1.025, 1.035)	< 0.001	1.021 (1.016, 1.027)	< 0.001	1.018 (1.013, 1.024)	< 0.001
DBP	1.037 (1.025, 1.048)	< 0.001	1.035 (1.023, 1.047)	< 0.001	1.028 (1.016, 1.040)	< 0.001
SUA	1.006 (1.005, 1.008)	< 0.001	1.004 (1.002, 1.006)	< 0.001	1.003 (1.000, 1.005)	0.018

Model 1: no adjust

Model 2: adjust for age, current smoking, current drinking

Model 3: adjust for age, current smoking, current drinking, BMI, FPG, TC, TG, HDL-C, LDL-C, eGFR

*Abbreviations*: *BMI* body mass index, *FPG* fasting plasma glucose, *TC* total cholesterol, *TG* triglyceride, *HDL-C* high-density lipoprotein cholesterol, *LDL-C* low-density lipoprotein cholesterol, *eGFR* estimated glomerular filtration rate, *SBP* systolic blood pressure, *DBP* diastolic blood pressure, *SUA* serum uric acid

Multivariate logistic regression was performed to reveal the association of hypertension and hyperuricemia with ischemic stroke by sex (Table 3). After adjusting for age, current smoking, current drinking, BMI, FPG, TC, TG, HDL-C, LDL-C and eGFR, hypertension was significantly associated with ischemic stroke in men (OR: 2.783, 95% CI: 1.793, 4.320) and in women (OR: 4.800, 95% CI: 2.945, 7.822). However, hyperuricemia was significantly associated with ischemic stroke only in women (OR: 1.888, 95% CI: 1.244, 2.864).

**Table 3:** Multivariate logistic regression of hypertension and hyperuricemia for ischemic stroke by sex

	Odds Ratio (95%CI)					
	Model 1	*P* value	Model 2	*P* value	Model 3	*P* value
Men						
Hypertension	4.700 (3.104, 7.116)	< 0.001	3.245 (2.119, 4.969)	< 0.001	2.783 (1.793, 4.320)	< 0.001
Hyperuricemia	1.386 (0.940, 2.042)	0.099	1.627 (1.092, 2.424)	0.017	1.431 (0.919, 2.230)	0.113
Women						
Hypertension	9.849 (6.186, 15.680)	< 0.001	5.933 (3.680, 9.566)	< 0.001	4.800 (2.945, 7.822)	< 0.001
Hyperuricemia	3.451 (2.413, 4.934)	< 0.001	2.529 (1.748, 3.661)	< 0.001	1.888 (1.244, 2.864)	0.003

Model 1: no adjust

Model 2: adjust for age, current smoking, current drinking

Model 3: adjust for age, current smoking, current drinking, BMI, FPG, TC, TG, HDL-C, LDL-C, eGFR

*Abbreviations*: *BMI* body mass index, *FPG* fasting plasma glucose, *TC* total cholesterol, *TG* triglyceride, *HDL-C* high-density lipoprotein cholesterol, *LDL-C* low-density lipoprotein cholesterol, *eGFR* estimated glomerular filtration rate

Table 4 shows logistic regression of the joint effect of hypertension and hyperuricemia for ischemic stroke by sex. Participants without both hypertension and hyperuricemia were defined as the reference group. For men, participants with hypertension and hyperuricemia had 5.9 times higher risk of ischemic stroke than those without them in model 1. After adjusting for age, current smoking, current drinking, BMI, FPG, TC, TG, HDL-C, LDL-C and eGFR (model 3), participants with both of them had 4.1 times higher risk than those without them in men. For women, participants with hypertension and hyperuricemia had 24.3 times higher risk of ischemic stroke than those without them in model 1. After full adjustment of covariates, participants with both of them had 8.9 times higher risk than those without them. Finally, the interaction between hypertension and hyperuricemia was statistically significant only in women rather than in men after full adjustment.

**Table 4:** Multivariate logistic regression of the joint effect of hypertension and hyperuricemia for ischemic stroke by sex

	Odds Ratio (95%CI)					
	Model 1	*P* value	Model 2	*P* value	Model 3	*P* value
Men						
HTN (−) HUA (−)	1 (reference)		1 (reference)		1 (reference)	
HTN (−) HUA (+)	2.516 (1.055, 6.000)	0.037	3.133 (1.299, 7.556)	0.011	2.890 (1.173, 7.121)	0.021
HTN (+) HUA (−)	5.498 (3.409, 8.866)	< 0.001	3.777 (2.317, 6.157)	< 0.001	3.296 (1.998, 5.437)	< 0.001
HTN (+) HUA (+)	5.917 (3.276, 10.688)	< 0.001	4.827 (2.642, 8.819)	< 0.001	4.059 (2.126, 7.751)	< 0.001
*P* value for interaction		0.007		0.011		0.073
Women						
HTN (−) HUA (−)	1 (reference)		1 (reference)		1 (reference)	
HTN (−) HUA (+)	2.136 (0.491, 9.284)	0.312	1.694 (0.387, 7.421)	0.484	1.353 (0.306, 5.991)	0.691
HTN (+) HUA (−)	8.956 (5.465, 14.677)	< 0.001	5.492 (3.309, 9.114)	< 0.001	4.592 (2.740, 7.696)	< 0.001
HTN (+) HUA (+)	24.254 (13.688, 42.976)	< 0.001	12.689 (7.027, 22.912)	< 0.001	8.913 (4.726, 16.809)	< 0.001
*P* value for interaction		< 0.001		< 0.001		< 0.001

Model 1: no adjust

Model 2: adjust for age, current smoking, current drinking

Model 3: adjust for age, current smoking, current drinking, BMI, FPG, TC, TG, HDL-C, LDL-C, eGFR

*Abbreviations*: *BMI* body mass index, *FPG* fasting plasma glucose, *TC* total cholesterol, *TG* triglyceride, *HDL-C* high-density lipoprotein cholesterol, *LDL-C* low-density lipoprotein cholesterol, *eGFR* estimated glomerular filtration rate

## Discussion

This study demonstrated the independent and positive correlations between hypertension, hyperuricemia and ischemic stroke in a rural Chinese population. More importantly, our study for the first time implicated the joint effect between hypertension and hyperuricemia towards ischemic stroke only in women, not in men. Our results suggest the joint effect of hypertension and hyperuricemia on ischemic stroke in women may be greater than the sum of their individual effects. Therefore, our research may provide a simple explanation for the public to understand the harm of hypertension and hyperuricemia on ischemic stroke.

In this study, hypertension was positively associated with the risk of ischemic stroke in both men and women, which was consistent with previous studies [21–23]. For a long time, elevated blood pressure has been associated with cardiovascular outcomes, and the correlation between hypertension and increased risk of stroke may be the strongest and easiest to recognize. Previous randomized controlled trials have suggested that lowering high blood pressure was positive treatment in patients with acute ischemic stroke [24, 25]. Evidence has shown that 80% of patients with acute ischemic stroke have hypertension, which is independently associated with poor prognosis [26–28].

Uric acid is a product of human purine metabolism and is known to be related to many risk factors for strokes, such as high blood pressure, obesity, and diabetes [13, 15, 29]. In our study, elevated uric acid was also positively associated with the risk of ischemic stroke but only in women. Consistently, the Rotterdam Study showed that elevated uric acid was a positive risk factor for stroke only in women [30]. However, some previous studies revealed the correlation between hyperuricemia and stroke in both men and women, though stronger in women than in men [31, 32]. In addition, elevated uric acid may modestly increase the risk of stroke morbidity and mortality [11, 12]. In this study, we adopted the classic threshold of 6 and 7 mg/dL SUA for women and men to define hyperuricemia. However, many recent studies indicate that a cardiovascular SUA threshold was significantly lower than that used for the classic definition of hyperuricemia [33, 34]. Thus, further researches are warranted to explore the combination of hypertension and hyperuricemia on ischemic stroke.

Both hypertension and hyperuricemia were positively associated with the risk of ischemic stroke. Results of our study also show that the combination of hypertension and hyperuricemia had more than nine-fold higher risk than those without hypertension and hyperuricemia in women. However, the joint effect of hypertension and hyperuricemia was not observed in men. These findings showed that the interaction between hypertension and hyperuricemia was statistically significant only in women rather than in men after adjustment for age, current smoking, current drinking, BMI, TG, HDL-C and eGFR. The potential mechanism related to ischemic stroke might be arterial stiffness caused by the combination of hyperuricemia and hypertension [35, 36].

There are some reasons that may explain the gender differences in the joint effect of hypertension and hyperuricemia to ischemic stroke. Previous studies have shown that hyperuricemia was more relevant with hypertension in women than men [37, 38]. In addition, the Apolipoprotein Mortality Risk Study suggested that uric acid was more strongly associated to stroke in women than in men [32]. Furthermore, according to a systematical review, individuals with moderate hypertension had a higher risk of stroke in women than in men [39]. Therefore, the gender differences in the joint effect of hypertension and hyperuricemia to ischemic stroke were reasonable.

This study still has several limitations that need to be noticed. Firstly, the cross-sectional design cannot prove the causality between hypertension, hyperuricemia, and ischemic stroke. Secondly, this study was conducted in rural areas of northeast China, which may produce selection bias. Thirdly, although a recent study showed that diuretic-related hyperuricemia had the same cardiovascular risk as nondiuretic-related hyperuricemia, the lack of information on diuretics may influence the outcome of serum uric acid and ischemic stroke in hypertensive patients [40]. Finally, we did not collect information about the history of stroke and related medicine.

## Conclusions

This study demonstrated the positive correlations between hypertension, hyperuricemia and ischemic stroke in a rural Chinese population. More importantly, our study demonstrated the joint effect between hypertension and hyperuricemia towards ischemic stroke only in women, not in men. Our results suggest the joint effect of hypertension and hyperuricemia on ischemic stroke in women may be greater than the sum of their individual effects.

## Data Availability

The datasets generated during and analysed during the current study are not publicly available due to individual privacy concerns but are available from the corresponding author on reasonable request.

## Abbreviations

SD: Standard deviation
BMI: Body mass index
FPG: Fasting plasma glucose
TC: Total cholesterol
TG: Triglyceride
HDL-C: High-density lipoprotein cholesterol
LDL-C: Low-density lipoprotein cholesterol
eGFR: Estimated glomerular filtration rate
SBP: Systolic blood pressure
DBP: Diastolic blood pressure
SUA: Serum uric acid
